# Prenatal Diagnosis of Keratitis–Ichthyosis–Deafness Syndrome With Dandy Walker Malformation: A Case Report

**DOI:** 10.1155/crog/5451804

**Published:** 2026-07-10

**Authors:** Lucas Bourdil, Carolin Georgia Blume

**Affiliations:** ^1^ Department of Gynaecology and Obstetrics, Cantonal Hospital of Graubünden, Lürlibadstrasse 118, 7000, Chur, Switzerland; ^2^ Department of Obstetrics and Gynecology, University Hospital Basel, Spitalstrasse 21, Basel, 4056, Switzerland, unispital-basel.ch

## Abstract

**Background:**

Keratitis‐ichthyosis‐deafness (KID) syndrome is a rare disorder characterized by progressive vascularizing keratitis, ichthyosiform erythrokeratoderma, and neurosensory hearing loss. It is caused by missense mutations in the GJB2 gene. Its known association with Dandy‐Walker malformation (DWM), a developmental anomaly of the posterior cranial fossa, has only been described once antenatally.

**Case Presentation:**

We report a case of a fetus with a DWM seen in neurosonography and fetal MRI. A whole exome sequencing revealed a heterozygous D50N mutation of the GJB2 gene, diagnosing KID syndrome. Postnatal findings demonstrated an erythrokeratoderma consistent with KID syndrome.

**Conclusion:**

This case supports a pathophysiologic link between D50N‐GJB2 mutations and DWM. It also highlights the importance of genetic testing in cases of isolated DWM. Further research is required to elucidate the pathophysiological mechanisms underlying the association between KID syndrome and DWM.

## 1. Introduction

The keratitis–ichthyosis–deafness (KID) syndrome is a rare disease characterized by progressive keratitis with corneal neovascularization, ichthyosiform scaling, palmoplantar hyperkeratosis, nail dystrophy, alopecia, and bilateral hearing loss [1]. Affected individuals are predisposed to severe infections and skin malignancies. KID syndrome results from missense mutations in the GJB2 gene, which encodes Connexin 26 (Cx26), a subunit of gap junction hemichannels. In ~80% of cases, KID syndrome is caused by a D50N mutation [2]. Other variants, such as A88V and G45E, are often lethal in infancy due to severe infections [3].

Dandy–Walker malformation (DWM) is defined by vermian hypoplasia, predominantly of the posterior lobe, an enlarged ponto‐vermian angle, an inferolateral displacement of the choroid plexus of the fourth ventricle, and an obtuse fastigial recess [4].

To date, there are only two reports of antenatal findings leading to the diagnosis of KID syndrome. In 2010, Sbidian et al. [5] have made the first antenatal molecular diagnosis of a lethal G45E mutation. Due to a maternal germline mutation, several siblings had already been affected, prompting early genetical testing in the reported pregnancy. In 2020, Okmen et al. [6] observed a DWM in a fetus that showed keratodermic lesions at birth, was postnatally diagnosed with an A88V mutation, and died of severe infection 40 days later.

We present a third case, where detection of DWM led to the antenatal molecular diagnosis of a D50N mutation.

## 2. Case Presentation

**Figure 1:**
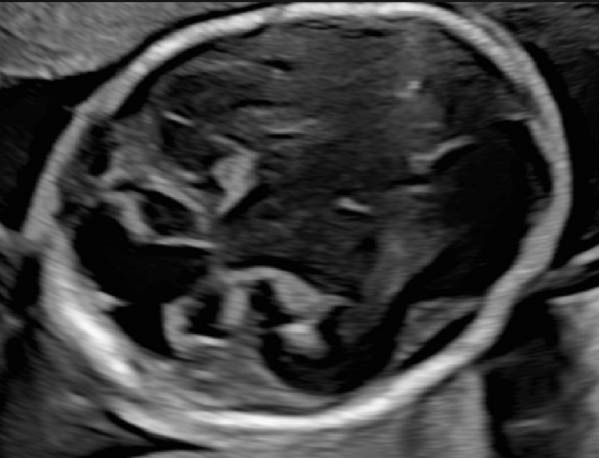
Abnormal posterior cranial fossa seen in the mid‐trimester on a transcerebellar axial plane.

During a routine mid‐trimester fetal morphological screening, an abnormal posterior fossa was revealed (Figure 1). A cystic gap between the cerebellar hemispheres was seen, suggesting a cranially rotated or hypoplastic vermis cerebellaris. Differential diagnoses included DWM, Blake’s pouch cyst (BPC), and cerebellar vermian hypoplasia (CVH). Our assessment was limited by poor imaging windows, only allowing an axial view on the posterior cerebral fossa.

**Figure 2:**
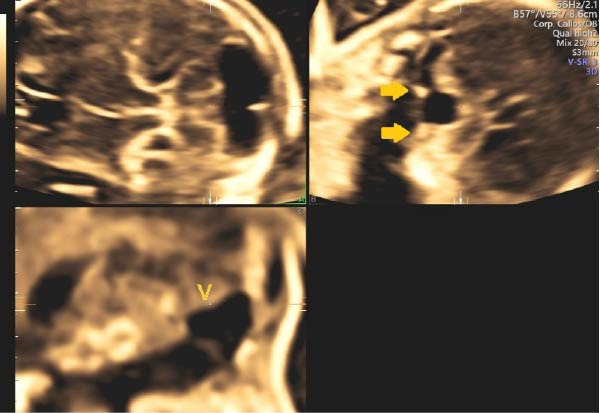
3D multiplanar representation of the posterior cranial fossa. Upper left: axial plane. Lower left: sagittal plane, with a hypoplastic, cranially rotated vermis (V) and a blunt fastigium recess. Upper right: coronal plane: the echogenic choroid plexus of the fourth ventricle (arrows) is latero‐caudally displaced, lying outside the fourth ventricle.

**Figure 3:**
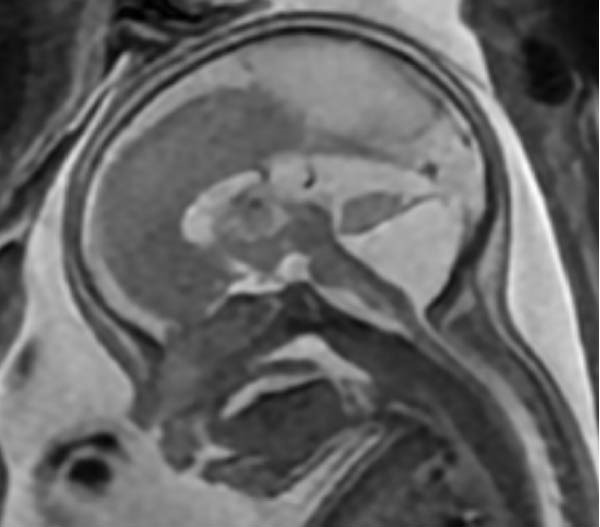
Fetal neuro‐MRI, T2‐sequence, showing a hypoplastic vermis, a broad ponto‐vermian angle, a blunt fastigium recess and a tail sign.

Signs consistent with DWM were later found using multiplanar image correlation (Figure 2). A hypoplastic vermis cerebellaris with a wide ponto‐vermian angle and a broad fastigium recess were seen on a sagittal plane. The coronal plane displayed a “down and out” position of the plexus choroideus of the fourth ventricle, which has been described by Paladini and Volpe as typical for DWM [7, 8]. A fetal magnetic resonance imaging confirmed the diagnosis of DWM (Figure 3).

An Amniocentesis was performed. A whole‐exome sequencing identified a heterozygous GJB2 mutation (c.148G>A; p.D50N), which is associated with KID syndrome. A uniparental mosaicism was also detected.

In order to respect the anonymity of the patient, who could not give written consent for publication after multiple failed attempts to contact her, we will not disclose any further details about the clinical management of this case. Postnatal examination of the fetus confirmed the presence of a generalized erythrokeratoderma, consistent with KID syndrome.

Given the uniparental mosaicism, the recurrence risk in future pregnancies could be as high as 50%. Prenatal diagnostic counseling was recommended for future conceptions.

## 3. Discussion

In the cochlea, Cx26 plays a crucial role in ion homeostasis and intercellular signaling required for cochlear maturation and auditory signal propagation. In fact, GJB2 mutations represent the most common genetic causes of prelingual hearing loss. The majority of these are recessive, nonsyndromic, loss‐of‐function mutations [9]. In contrast, KID syndrome results from gain‐of‐function dominant mutations affecting the extracellular loop (EL1) of Cx26 by impairing its gating function. We know from in vitro studies that at least two variants (D50N and G11E) of these leaky hemichannels induce cell death through altered calcium homeostasis [10]. This could explain the skin manifestations of KID syndrome, as disrupted calcium signaling can lead to premature keratinocyte apoptosis and hyperkeratinisation [11].

In 2009, Todt et al. [12] reported DWM in eight out of nine patients with KID syndrome carrying the D50N variant, as observed in our case, suggesting that this association is not fortuitous. DWM has also been described in association with the A88V variant [6]. To date, the pathophysiology of this association remains unexplained. Recently, a case of CVH was reported in a deaf individual with a nonsyndromic GJB2 mutation, further supporting a role for GJB2 in vermian development [13].

Haldipur et al. [14] have proposed a unified pathogenetic model for DWM and CVH: an early insult to the developing rhombic lip prior to its internalization into the vermis at 14 postconceptional weeks leads to DWM, whereas a later insult leads to CVH. In mice, Cx26 hemichannels are essential for cortical neuronal migration by attaching migrating neurons to a radial glial scaffold [15, 16]. It remains uncertain whether GJB2‐associated vermian malformations result from an impaired neuronal migration within the developing vermis or from a primary Cx26‐mediated disturbance of the rhombic lip during early cerebellar development.

Interestingly, all patients with KID syndrome and DWM reported by Todt et al. [12] exhibited minimal or no neurological abnormalities. This is unexpected, as neurodevelopmental impairment occurs in ~45 % of cases of isolated DWM [17]. In isolated DWM, the neurological outcome may correlate more closely with the nature and extent of the underlying developmental insult than with the anatomical malformation itself. We therefore hypothesize that the DWM‐associated insult in KID syndrome may be relatively restricted, potentially explaining the favorable neurodevelopmental outcomes observed in reported cases.

In families affected by KID syndrome, early targeted genetic testing should be offered when the diagnosis has potential implications for the expectant parents. Detection of posterior fossa abnormalities consistent with DWM may serve as a prenatal indicator prompting molecular investigation. More generally, in cases of apparently isolated DWM, the reported diagnostic yield of karyotyping, chromosomal microarray, and next‐generation sequencing is 10.7%, 15.4%, and 16.5%, respectively [17]. Hence, we advocate maintaining a low threshold for proposing genetic testing, including whole‐exome sequencing, in fetuses with isolated DWM.

## 4. Conclusion

We report a case of antenatally diagnosed KID syndrome, confirmed by whole‐exome sequencing following the detection of DWM. Further research is required to elucidate the pathophysiological mechanisms underlying the association between KID syndrome and DWM.

In fetuses with apparently isolated DWM, we recommend proposing genetic testing, including whole‐exome sequencing. In fetuses at risk of KID syndrome, early sonographic assessment of the posterior cranial fossa may be informative, but molecular testing is essential for accurate diagnosis and adequate counseling.

## Funding

No funding was received for this manuscript.

## Conflicts of Interest

The authors declare no conflicts of interest.

## Data Availability

The data that support the findings of this study are available upon request from the corresponding author. The data are not publicly available due to privacy or ethical restrictions.
